# Association of matrix metalloproteinase 7 and the alpha-chain of fibrinogen at baseline with response to methotrexate at 3 months in patients with early rheumatoid arthritis

**DOI:** 10.1186/s41927-025-00509-8

**Published:** 2025-05-21

**Authors:** Karen Hambardzumyan, Carl Hamsten, Lucía Lourido, Saedis Saevarsdottir, Peter Nilsson, Ronald F. van Vollenhoven, Per-Johan Jakobsson, Helena Idborg

**Affiliations:** 1 Division of Rheumatology, Department of Medicine, Center for Molecular Medicine, Karolinska Institutet, Karolinska University Hospital, Karolinska University Hospital, Solna, Stockholm, SE-17176 Sweden; 2 Unit of Immunology and Allergy, Department of Medicine, Karolinska Institutet and Karolinska University Hospital, Stockholm, Sweden; 3 Grupo de Investigación de Reumatología (GIR) - Unidad de Proteómica, Instituto de Investigación Biomédica de A Coruña (INIBIC), Complexo Hospitalario Universitario de A Coruña (CHUAC), Sergas, A Coruña, Spain; 4 Division of Clinical Epidemiology, Department of Medicine Solna, Karolinska Institutet, Stockholm, Sweden; 5 Faculty of Medicine, School of Health Sciences, University of Iceland, Reykjavik, Iceland; 6 Affinity Proteomics, SciLifeLab, School of Biotechnology, KTH – Royal Institute of Technology, Stockholm, Sweden; 7 Amsterdam Rheumatology and Immunology Center, Amsterdam, The Netherlands

**Keywords:** Rheumatoid arthritis, Biomarkers, Prediction, Affinity proteomics, Methotrexate therapy, Clinical response

## Abstract

**Background:**

The identification of responders to methotrexate (MTX) would optimize the therapy of patients with early rheumatoid arthritis (eRA). Our aim was to identify protein biomarkers for the prediction of the response to MTX.

**Methods:**

We analysed patients with eRA (*N* = 135) from the Swedish Pharmacotherapy (SWEFOT) trial population (Trial registration number: NCT00764725). Baseline serum levels of 177 proteins with an inflammatory signature were profiled via 380 antibodies in a suspension bead array format. Protein levels were analysed for their associations with the achievement of a low 28-joint disease activity score (LDA = DAS28 ≤ 3.2) after 3 months of MTX therapy (primary outcome) or a good response according to the European Alliance of Associations for Rheumatology (EULAR) criteria (secondary outcome).

**Results:**

Multivariable analysis revealed that the serum levels of two of the 177 proteins at baseline, matrix metalloproteinase 7 (MMP-7) and the alpha-chain of fibrinogen (FGA), were significantly different between patients who did and did not achieve LDA at 3 months. Among patients with low versus high levels of either MMP-7 or FGA, 60% versus 24% and 58% versus 22%, respectively, achieved LDA (*p* < 0.001). Among patients with low levels of both proteins, 79% achieved LDA at 3 months, whereas only 18% of those with high levels of both proteins achieved LDA at 3 months (*p* < 0.001). The results were similar when a secondary outcome was used.

**Conclusions:**

Low levels of MMP-7 and FGA at baseline were associated with improved clinical outcomes in eRA patients. Validation of these results in another eRA cohort is now warranted, and if confirmed, it may facilitate clinical decision-making regarding whether to start with MTX in monotherapy or more potent alternatives.

**Supplementary Information:**

The online version contains supplementary material available at 10.1186/s41927-025-00509-8.

## Introduction

Rheumatoid arthritis (RA) is a chronic inflammatory disease that primarily affects joints [1]. Methotrexate (MTX), among the available disease-modifying antirheumatic drugs (DMARDs), is the standard first choice for the treatment of patients with early rheumatoid arthritis (eRA) [2, 3]. However, only 30–40% of RA patients on MTX achieve low disease activity [4, 5]. Predictive measures would be much appreciated in clinical practice to identify responders/nonresponders to MTX therapy, making it possible to modify treatment at an earlier stage and limiting persistent disease activity and the risk of long-term disability.

Previous studies have evaluated predictive factors for the response to MTX, some with consistent results and some with controversial results. For example, among demographic factors, female sex, younger age and current smoking status have been shown by many studies, including the Swedish Farmacotherapy (SWEFOT) trial, to be predictors of poor response to MTX [6–8]. For protein biomarkers as predictors of MTX efficacy, the data are less consistent. For example, some studies, including SWEFOT, could not demonstrate any predictive value of autoantibodies for subsequent response to MTX [6, 7]. In contrast, in the PROMPT trial, anti-CCP positivity was associated with improved clinical outcomes [9]. Potential biomarkers for the response to MTX therapy have recently been reviewed [10], but have not been validated and implemented in clinical practice [11]. 

In the present study, we investigated a panel of 177 proteins with an inflammatory signature for the prediction of the response to MTX in eRA patients from the SWEFOT trial.

## Methods

### Patient population

The SWEFOT trial (Trial registration number: NCT00764725, Study start 2002-12, Study completion 2008-12, https://www.clinicaltrials.gov/) has been initiated to compare treatment efficacy between a combination of conventional synthetic DMARD therapy (triple therapy) and biological therapy in MTX-inadequate responders [4]. The trial was conducted in accordance with the Declaration of Helsinki and was approved by the regional ethics committee. All patients provided written informed consent before enrolment in the trial.

Among the 493 DMARD-naïve, clinically active (28-joint disease activity score [DAS28] > 3.2) eRA patients recruited for the SWEFOT trial, 135 patients were analysed at baseline, 85 of whom did not achieve low disease activity (LDA = DAS28 ≤ 3.2) after 3 months of MTX monotherapy. The 135 patients were randomly selected, and then the selection was adjusted with the aim of achieving an even distribution of autoantibody positivity among men and women, since rheumatoid factor (RF) positivity might influence the detection of certain proteins. With this type of selection, it is possible to evaluate if a potential biomarker is relevant only in RF-positive or RF-negative patients. However, the selection will not be representative of the SWEFOT cohort since the proportion of RF-positive patients will be greater in the selection cohort, and RF positivity is associated with more severe disease.

The primary outcome was the achievement of LDA after 3 months of MTX monotherapy. The secondary outcome was a good response based on the European Alliance of Associations for Rheumatology (EULAR) criteria [12]. 

### Suspension bead arrays

Target proteins were selected based on a combination of literature review and expert input, focusing on those proteins previously associated with rheumatoid arthritis and inflammatory pathways. A total of 380 antibodies covering 177 target proteins were obtained from the Human Protein Atlas (Supplementary Table S1) and were immobilized on color-coded magnetic beads as previously described [13]. Four quality controls were included; antihuman IgG (Jackson ImmunoResearch) and antialbumin (Dako) were used as positive controls, and rabbit IgG from nonimmunized rabbits (Bethyl) and bead identity with no proteins immobilized were used as negative controls. All the serum samples were randomized into 96-well plates, diluted 1:10 and labelled with biotin as previously described [14]. Proteins were detected by incubation with a streptavidin-conjugated fluorophore followed by readout in a FlexMap3D instrument (Luminex Corp.). Proteins with missing data > 20% in a subgroup were excluded from the analysis. The results are reported as the median fluorescent intensity (MFI) per bead identity and sample, and no further preprocessing of data, e.g., transformation or centering, was performed before further data analysis.

### Statistical analysis

The levels of the 177 target proteins for the 380 antibodies at baseline were compared between patients who achieved LDA and those who did not achieve LDA after 3 months of MTX monotherapy via the Mann‒Whitney U test. For eight proteins, p values < 0.001 were obtained. After applying the Bonferroni correction, these proteins were not considered to differ significantly (*p* > 0.0001). The Bonferroni-adjusted significance threshold is 0.05 / 380 = 0.00013. If a less stringent approach, such as the Benjamini-Hochberg correction, had been applied, the significance cut-off for the 8th-ranked protein would have been approximately 0.00105. However, although these eight proteins did not reach statistical significance in the univariate analysis after Bonferroni correction, they were considered potential candidates for biomarkers and were subsequently analysed in a multivariable logistic regression model. The univariate analysis was only used to select factors/proteins to be included in the regression model, and only independent predictors (*n* = 2 of the 8 candidate proteins) were chosen for further analysis. The logistic regression model was only adjusted for age and sex as potential confounders. RF-positivity was adjusted for by means of the correction of the selection of patients. The independent predictors were analysed via receiver operating characteristic (ROC) curve analysis to define the area under the curve (AUC) and cut-off, which was based on the highest sum of sensitivity with specificity. Using the generated cut-offs, the patients were dichotomized, and the proportions of patients who achieved LDA or EULAR good response were compared between the groups via the chi-square test. Statistical analyses were performed via IBM SPSS version 24.

## Results

### Patient characteristics and protein levels at baseline

Among the 135 patients included in this study, compared with the remaining 358 patients from the SWEFOT trial, four baseline parameters (RF positivity, ESR, CRP and pain VAS) differed significantly (Table 1), but the group was otherwise representative of the trial as a whole.

**Table 1:** Baseline characteristics and demographic data of patients from the SWEFOT trial

Baseline Characteristics^a^	Patients not analysed by AP (*N* = 358)^b^	Patients analysed by AP (*N* = 135)^c^	*P*- value^d^
Female: N (%)	246 (69)	101 (75)	0.186
Age (years)	57 (46–64)	57 (45–65)	0.860
Current Smokers: N (%)	61 (26)	26 (22)	0.363
Symptom Duration (months)	5 (3–8)	5 (4–8)	0.991
Anti-CCP-positive: N (%)	197 (66)	79 (58)	0.128
RF-positive: N (%)	256 (72)	78 (58)	**0.002**
28 Swollen Joint Count	10 (7–14)	10 (6–15)	0.936
28 Tender Joint Count	9 (5–13)	9 (5–13)	0.995
ESR (mm/h)	35 (22–56)	31 (17–47)	**0.019**
CRP Level (mg/L)	21 (9–47)	15 (8–35)	**0.014**
PatG (VAS 0–100 mm) Score	59 (40–75)	56 (35–72)	0.156
Pain VAS	59 (42–73)	52 (38–68)	**0.038**
HAQ	1.13 (0.82–1.63)	1.0 (0.75–1.50)	0.074
DAS28	5.8 (5.1–6.4)	5.6 (4.9–6.3)	0.104

AP – affinity proteomics, IQR – interquartile range, anti-CCP - anti-cyclic citrullinated peptide, RF – rheumatoid factor, ESR – erythrocyte sedimentation rate, CRP – C-reactive protein, PatG – Patient’s Global Assessment of Disease Activity, VAS – visual analogue scale, HAQ – health assessment questionnaire, DAS – disease activity score

^a^ Data are presented in median (IQR), unless otherwise mentioned; ^b^ Number of patients whose information is missing for “Patients not included” column: Current Smokers (*n* = 124), Anti-CCP (*n* = 60), RF and HAQ (*n* = 5), Swollen and Tender joint count (*n* = 2), ESR (*n* = 4), CRP, PatG VAS and Pain VAS (*n* = 3) and DAS28 (*n* = 7); ^c^ Number of patients whose information is missing for “Analysed Patients” column: Current Smokers (*n* = 15), ESR, PatG VAS, Pain VAS and HAQ (*n* = 1); DAS28 (*n* = 2); ^d^ p-value represent probability of null hypothesis being correct for Mann-Whitney U test (for continues variables) or Chi-squared test (for categorical variables)

After adjustment for multiple testing (Bonferroni correction, *p* = 0.05/380 tests = 0.0001), no serum proteins differed significantly between patient groups who achieved (*N* = 50) and did not achieve (*N* = 85) LDA at 3 months. A threshold of *p* < 0.001 was used for a more generous selection of potential candidates for prediction for further multivariable analysis. Among the 177 serum proteins, the baseline levels of the following proteins differed between the two groups (*p* < 0.001): interferon regulatory factor 8 (IRF8), C-C motif chemokine ligand 11 (CCL11), interleukin-17B receptor (IL-17RB), regulator of G-protein signalling (RGS18), oxidized low-density lipoprotein receptor 1 (OLR1), matrix metalloproteinase 7 (MMP-7), α-chain of fibrinogen (FGA) and complement component 8 γ-subunit (C8G) (Supplementary Figure S1).

For the detection of most of the target proteins, more than one antibody was used (177 proteins targeted by 380 antibodies) (Supplementary Table S1). Three antibodies against MMP-7 and two antibodies against FGA had correlation coefficients of 0.64 and 0.74, respectively.

### Associations of baseline categories of MMP-7 and FGA with clinical outcome at 3 months

**Fig. 1:**
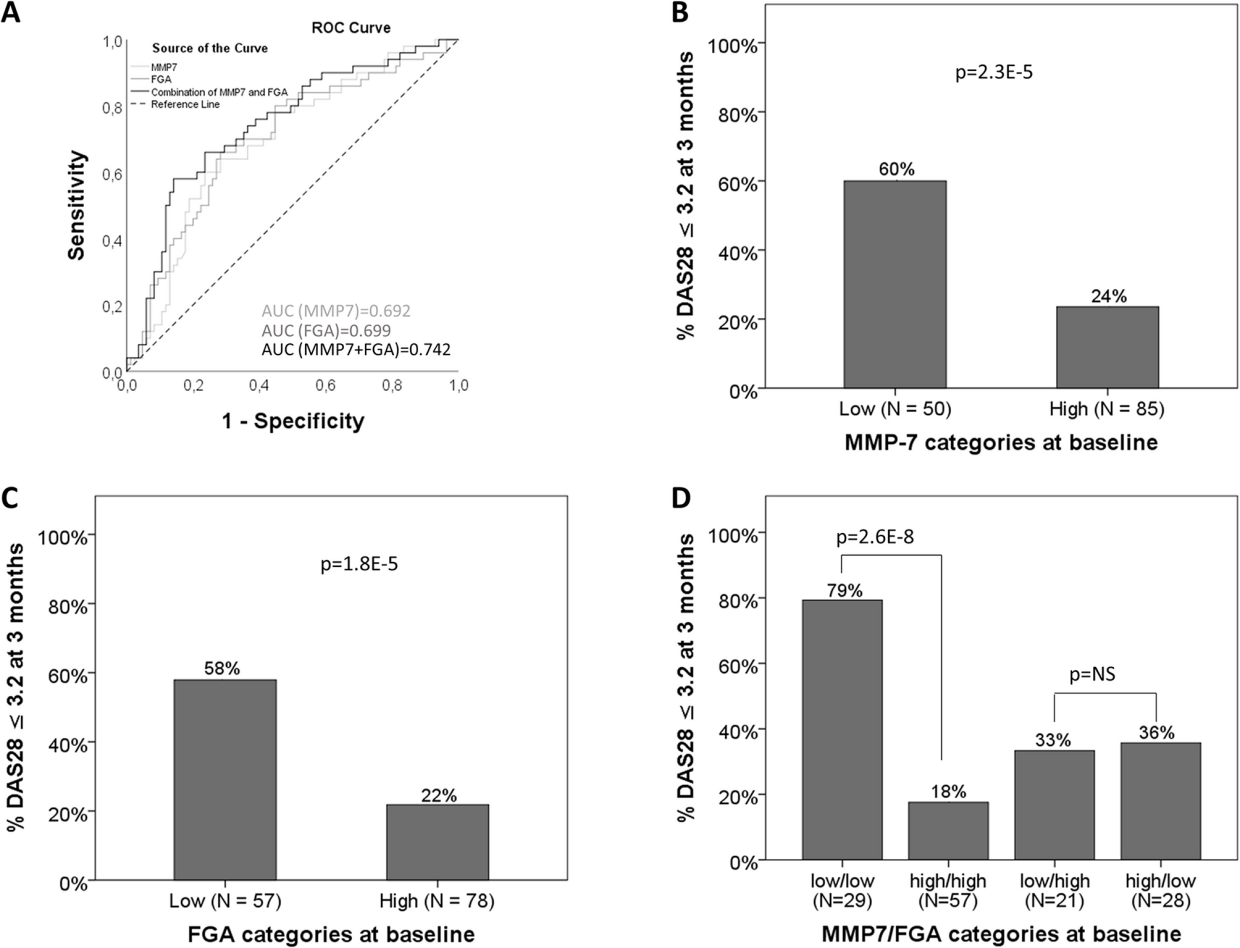
Assessment of baseline levels of MMP-7 and FGA as predictors of LDA at 3 months. Receiver operating characteristic curve analysis and area under the curve (**A**) of MMP-7 (light gray line), FGA (dark gray line) and the combination of MMP-7 and FGA (black line); proportion of patients who achieved a low DAS28 at 3 months among the groups dichotomized by MMP-7 (**B**), FGA (**C**) or the combination of MMP-7 and FGA (**D**)

In stepwise multivariable logistic regression analysis of the 8 proteins mentioned above, low baseline levels of MMP-7 and FGA were independently associated with the achievement of LDA after 3 months of MTX monotherapy, and continuous values of MMP-7 and FGA at baseline were analysed by ROC curve analysis, revealing AUCs of 0.692 (*p* < 0.001, 95% CI: 0.601–0.783) and 0.699 (*p* < 0.001, 95% CI: 0.606–0.792), respectively. ROC analysis of the combined MMP-7 and FGA data yielded an AUC of 0.742 (*p* < 0.001, Fig. 1A).

60% of patients with low MMP-7 levels at baseline (*N* = 50) achieved LDA after 3 months of MTX monotherapy, whereas only 24% of those with high MMP-7 levels (*N* = 85) achieved LDA (*p* < 0.001, Fig. 1B). Similar proportions were observed when patients with low (*N* = 57) versus high (*N* = 78) FGA levels were compared (58% versus 22%, *p* < 0.001; Fig. 1C).

**Fig. 2:**
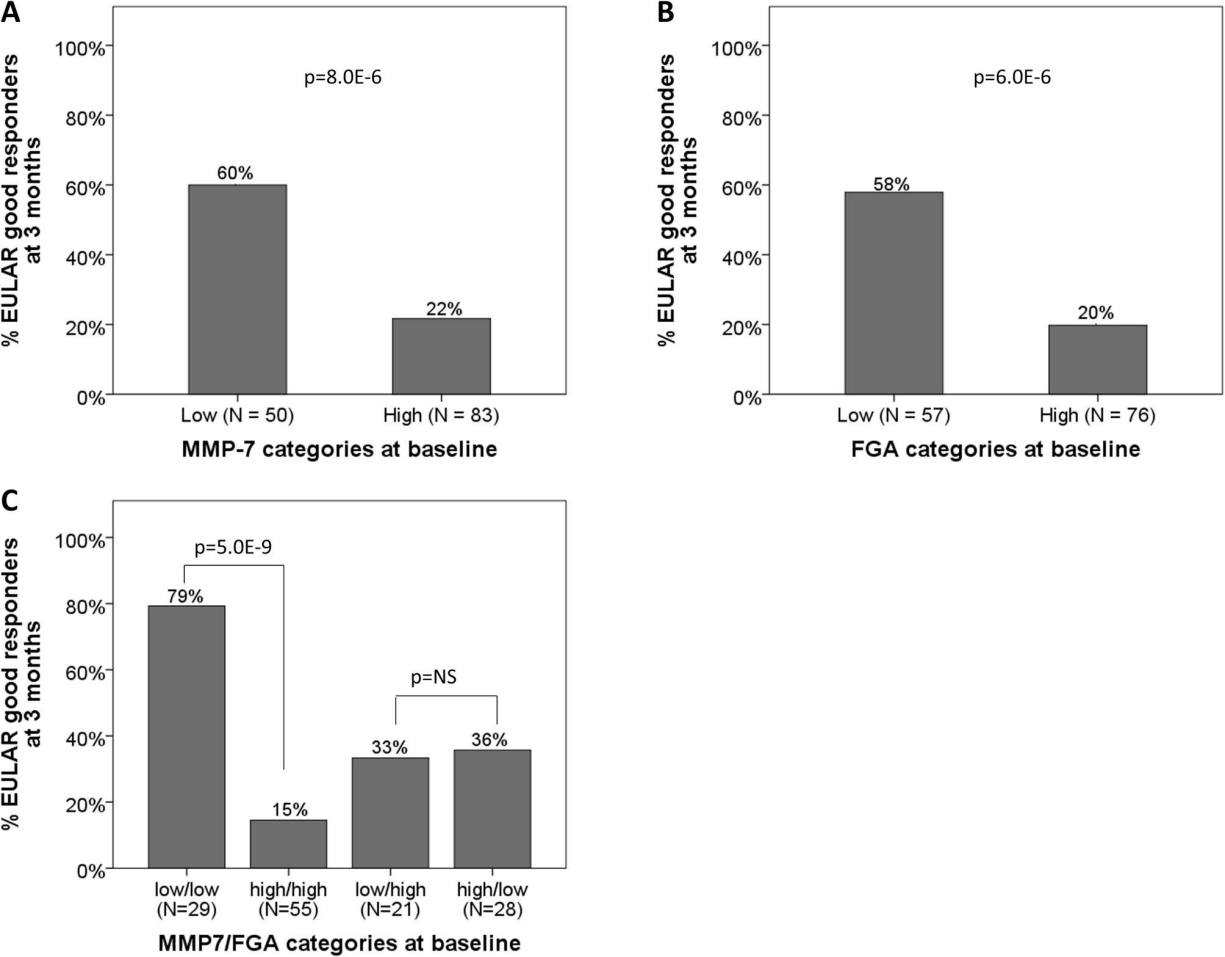
Proportion of MTX responders among patients with low versus high levels of serum biomarkers. Proportion of patients with good EULAR response among those dichotomized by MMP-7 (**A**), FGA (**B**) or a combination of MMP-7 and FGA (**C**)

79% (*n* = 23/29) of patients with low levels of both MMP-7 and FGA at baseline achieved LDA at 3 months, whereas only 18% (*n* = 10/57) of patients with high levels of both proteins did so (*p* < 0.001, Fig. 1D). Patients with low levels of MMP-7 and high levels of FGA (*n* = 21), as well as those with high levels of MMP-7 and low levels of FGA (*n* = 28), did not differ significantly in terms of the proportion of patients who achieved LDA: 33% and 36%, respectively (*p* = 0.862, Fig. 1D). Similar results were observed for good EULAR response as a treatment outcome (60% versus 22%, *p* < 0.001; 58% versus 20%, *p* < 0.001; and 79% versus 15%, *p* < 0.001, respectively; Fig. 2A, B and C). These comparisons of proportions with LDA or EULAR good responses within each stratum of the baseline DAS28 (moderate or high) resulted in a similar pattern (Supplementary Figure S2).

## Discussion

In this post hoc analysis of eRA patients who participated in the SWEFOT trial, we studied a panel of 177 serum proteins at baseline, prior to the initiation of DMARDs, to predict the response to subsequent 3 months of MTX monotherapy. Among the strengths of this trial is that the patients were DMARD treatment naïve (only allowing the use of low-dose corticosteroids if the dose had been stable for > 4 weeks), with few inclusion and exclusion criteria, thereby representing a typical early RA population [4, 15]. 

We found that two proteins (MMP-7 and FGA) were associated with the achievement of good LDA or EULAR responses at 3 months, with clinically meaningful differences between the groups. Unlike other MMPs, MMP-7 has a broader proteolytic function. Kazantseva et al. demonstrated that higher expression of MMP-7 was associated with a specific polymorphism in the MMP-7 promoter and was greater in extra-articular subcutaneous rheumatoid nodules than in the synovium [16]. They also demonstrated that in nodules of patients with higher expression of MMP-7, there was more fibrinoid collagen and infiltrated neutrophils than in nodules with lower expression of MMP-7.

Fibrinogen accumulates in the inflamed joints of patients with RA. Its citrullination (both α- and β-chains) is a target for autoantibodies [17, 18], which are associated with more destructive disease. Since MTX is not known for its ability to reduce citrullination or influence fibrinogen levels, patients with lower FGA levels at baseline are presumably more likely to respond, as evidenced by the results.

We could not find any published data concerning the ability of MMP-7 or FGA to predict the clinical response to MTX. Therefore, validation of these results is highly relevant.

The strengths of this study were the inclusion criteria of the SWEFOT trial, which allowed us to assess MTX effectiveness in DMARD-naïve eRA patients. Another strength is the large number of proteins screened, the choice of which was based on ongoing inflammatory and autoimmunity studies. One limitation, but also a strength, was that patients were selected to have similar RF-positivity in responders and non-responders groups. The baseline CRP, ESR and VAS pain differed at baseline between the two groups which might contribute to the differences between the groups. However, these factors might also be part in predicting the response.

There were several limitations as well. The cut-offs for MMP-7 and FGA that are based on the MFI need to be validated on the basis of concentration. Next, the SWEFOT trial was not designed to investigate these biomarkers for the prediction of the response to MTX. Therefore, the selected patients for this study, which were selected with the aim of achieving an even distribution of autoantibody positivity among men and women since RF positivity might influence the detection of certain proteins, were not representative of the entire SWEFOT; consequently, it is not possible to interpret previously published predictors [6] in this subset.

## Conclusions

In summary, two of the 177 proteins measured prior to DMARD therapy in early RA, MMP-7 and FGA, were associated with the response to MTX at 3 months. A greater proportion of patients with low levels of these proteins were responders than those with high levels of MMP-7 and FGA. The differences were clinically meaningful, and the method, after minor modifications, is feasible in a routine clinical setting. Validation of these results in another eRA cohort using the same detecting antibodies is now an important first validation step toward eventual implementation to optimize early RA treatment.

## Electronic supplementary material

Below is the link to the electronic supplementary material.

Supplementary Material 1

Supplementary Material 2

Supplementary Material 3

Supplementary Material 4

## Data Availability

The datasets used and/or analysed during the current study are available from the corresponding author upon reasonable request.
