# Supplementary figures and images for: Association of matrix metalloproteinase 7 and the alpha-chain of fibrinogen at baseline with response to methotrexate at 3 months in patients with early rheumatoid arthritis
Source: BMC Rheumatol. 2025 May 21;9:56. doi: 10.1186/s41927-025-00509-8 (PMC12093799; doi:10.1186/s41927-025-00509-8)

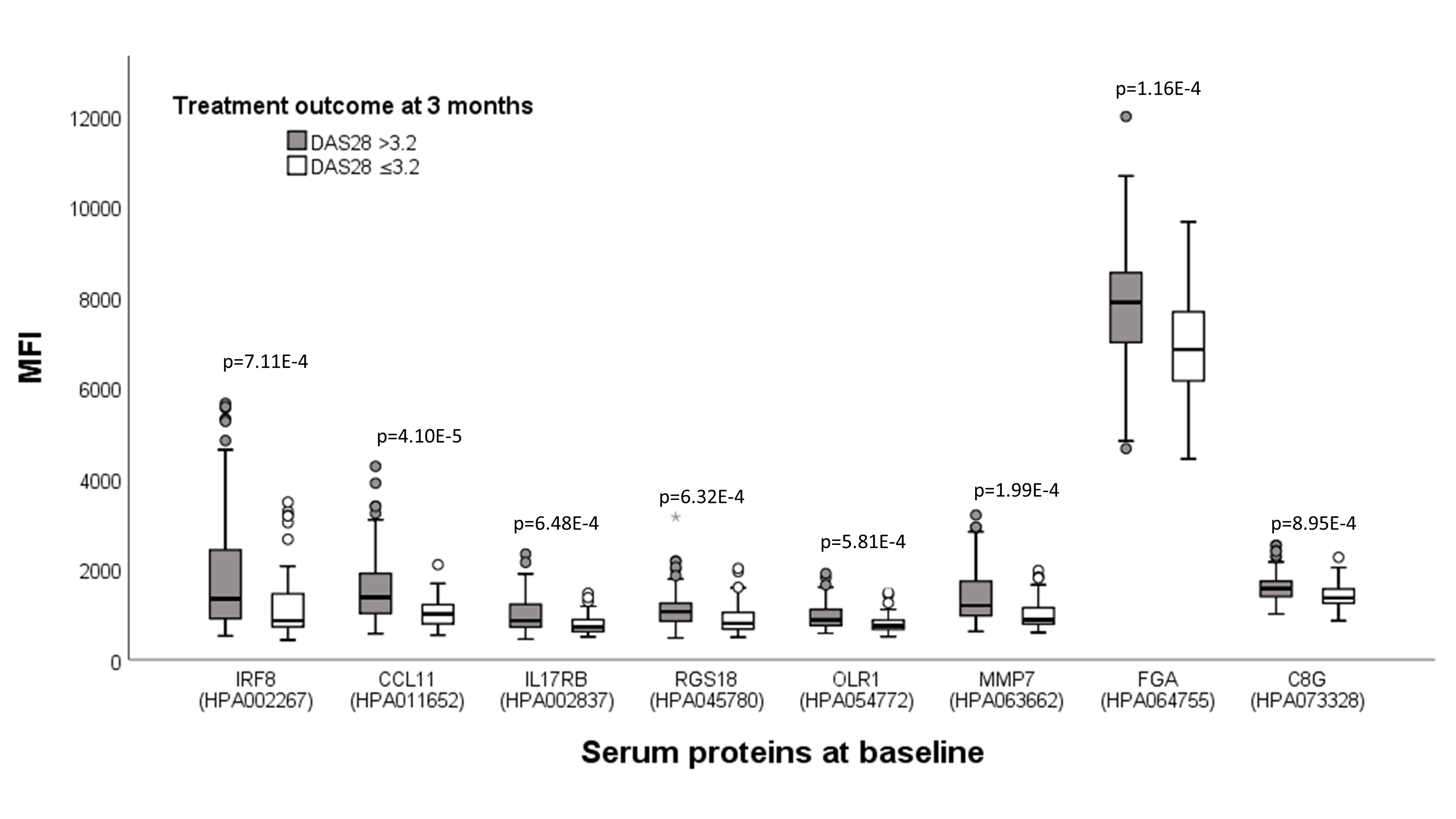

Supplement: Supplementary file 1 — Supplementary Material 1 [file 41927_2025_509_MOESM1_ESM.tif]

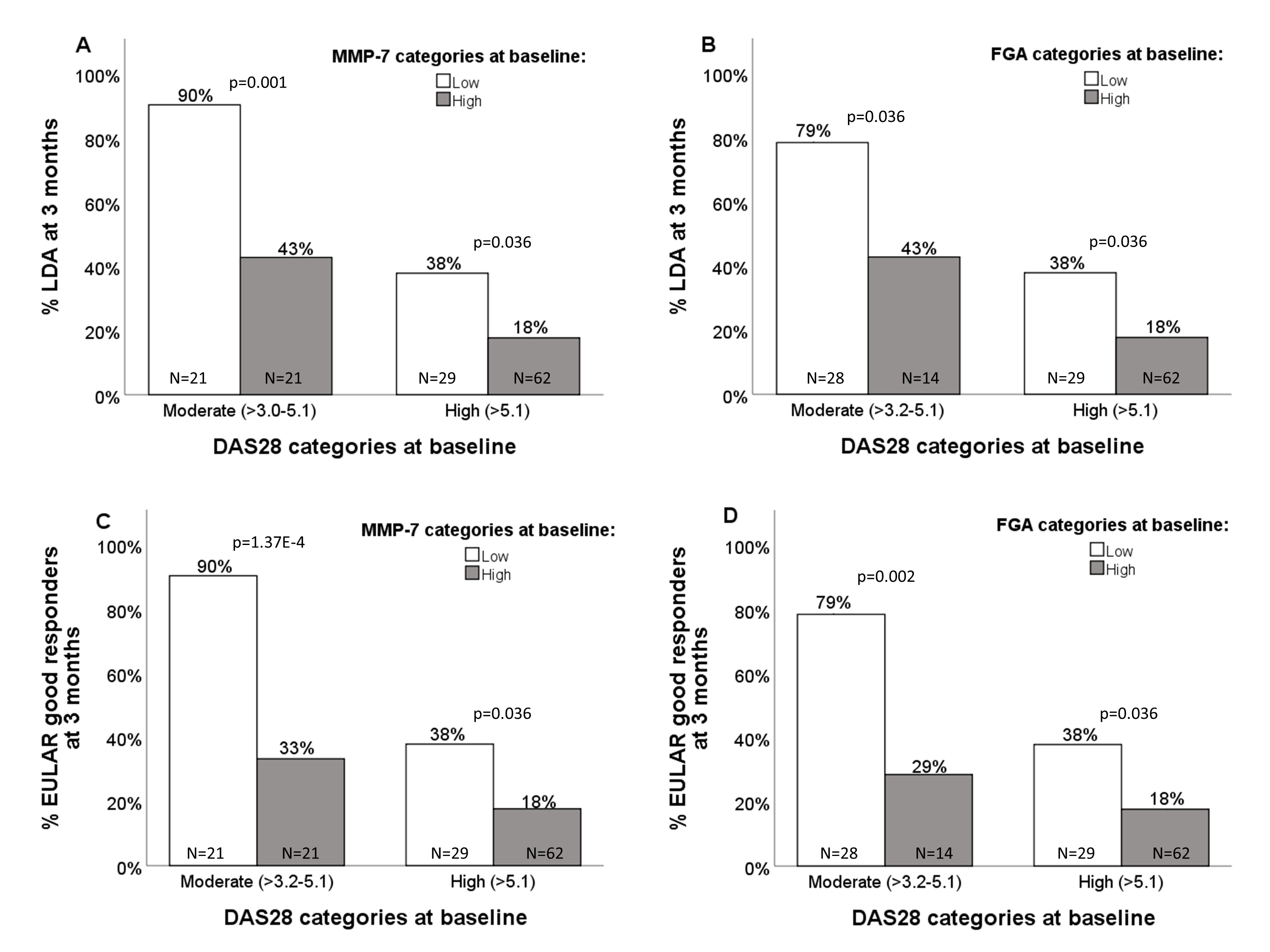

Supplement: Supplementary file 2 — Supplementary Material 2 [file 41927_2025_509_MOESM2_ESM.tif]
