# Supplementary material for: Association of matrix metalloproteinase 7 and the alpha-chain of fibrinogen at baseline with response to methotrexate at 3 months in patients with early rheumatoid arthritis
Source: BMC Rheumatol. 2025 May 21;9:56. doi: 10.1186/s41927-025-00509-8 (PMC12093799; doi:10.1186/s41927-025-00509-8)
Supplement: Supplementary file 3 — Supplementary Material 3 [file 41927_2025_509_MOESM3_ESM.docx]

| **Supplementary Table S1. ID numbers and targets of all antibodies (n=380) used in this study as well as four controls** | | | | | | | | | | | | | |
| --- | --- | --- | --- | --- | --- | --- | --- | --- | --- | --- | --- | --- | --- |
| **Abtibody ID** | **Targets** |  | **Abtibody ID** | **Targets** |  | **Abtibody ID** | **Targets** |  | **Abtibody ID** | **Targets** |  | **Abtibody ID** | **Targets** |
| HPA002265 | A2M |  | HPA054392 | CRISP3 |  | HPA005772 | IL1R2 |  | HPA047236 | NAPSA |  | HPA001823 | SMPD1 |
| HPA042506 | ACER1 |  | HPA048058 | CSF2 |  | HPA027598 | IL1R2 |  | HPA047744 | NAPSA |  | HPA018125 | SMPD2 |
| HPA013778 | ACER2 |  | HPA057404 | CSF2 |  | HPA007406 | IL1RL1 |  | HPA051835 | NCOA2 |  | HPA044442 | SMPD3 |
| HPA014092 | ACER2 |  | HPA071579 | CSF2 |  | HPA007917 | IL1RL1 |  | HPA069172 | NCOA2 |  | HPA058722 | SMPD3 |
| HPA038015 | ADCY2 |  | HPA040361 | CX3CL1 |  | HPA053829 | IL25 |  | HPA008422 | NFKB2 |  | HPA065535 | SMPD3 |
| HPA038483 | ADCY2 |  | HPA056729 | CX3CL1 |  | HPA068341 | IL25 |  | HPA050298 | NGF |  | HPA069383 | SMPD3 |
| HPA064436 | AGER |  | HPA073943 | CX3CL1 |  | HPA055164 | IL26 |  | HPA063135 | NGF |  | HPA001814 | SOD2 |
| HPA069474 | AGER |  | HPA014763 | DEGS1 |  | HPA061959 | IL26 |  | HPA003871 | NOS2 |  | HPA067587 | SPARCL1 |
| AntiAlbumin | Positive control |  | HPA014971 | DEGS1 |  | HPA069269 | IL26 |  | HPA038086 | NOS2 |  | HPA067641 | SPARCL1 |
| AntihumanIgG | Positive control |  | HPA057124 | DEGS1 |  | HPA046738 | IL2RA |  | HPA046487 | NOS2 |  | HPA022829 | SPHK1 |
| HPA046964 | ANXA2 |  | HPA021296 | DLG2 |  | HPA054622 | IL2RA |  | HPA007048 | NPSR1 |  | HPA023674 | SPHK1 |
| HPA061798 | ANXA2 |  | HPA021307 | DLG2 |  | HPA008412 | IL3 |  | HPA007106 | NPSR1 |  | HPA028761 | SPHK1 |
| HPA046715 | APOA1 |  | HPA023896 | DLG2 |  | HPA030770 | IL3 |  | HPA007489 | NPSR1 |  | HPA067105 | SPHK1 |
| HPA001352 | APOA4 |  | HPA027241 | ECM1 |  | HPA066598 | IL3 |  | HPA007976 | NPSR1 |  | HPA049062 | SPHK2 |
| HPA056395 | APOC3 |  | HPA066836 | ELANE |  | HPA022899 | IL33 |  | HPA050212 | NR3C1 |  | HPA057659 | SPHK2 |
| HPA065365 | APOC3 |  | HPA073774 | ELANE |  | HPA024426 | IL33 |  | HPA035619 | OLR1 |  | HPA065508 | SPHK2 |
| HPA065539 | APOE |  | HPA050507 | EPX |  | HPA052386 | IL33 |  | HPA035620 | OLR1 |  | HPA005562 | SPP1 |
| HPA068768 | APOE |  | HPA004826 | F7 |  | HPA007714 | IL4 |  | HPA050798 | OLR1 |  | HPA027541 | SPP1 |
| HPA001654 | APOH |  | HPA063808 | F7 |  | HPA063382 | IL4 |  | HPA054772 | OLR1 |  | HPA024330 | SPRR3 |
| HPA003732 | APOH |  | HPA035132 | FETUB |  | HPA070010 | IL4 |  | HPA047725 | ORM1.ORM2 |  | HPA044467 | SPRR3 |
| HPA047382 | ARFGAP1 |  | HPA035133 | FETUB |  | HPA065029 | IL5 |  | HPA057726 | ORM1.ORM2 |  | HPA044247 | SPTLC3 |
| HPA051019 | ARFGAP1 |  | HPA069860 | FETUB |  | HPA001325 | IL6 |  | HPA011325 | PDGFB |  | HPA048079 | SPTLC3 |
| HPA056273 | ARFGAP1 |  | HPA051370 | FGA |  | HPA044648 | IL6 |  | HPA011972 | PDGFB |  | HPA062197 | SPTLC3 |
| HPA040622 | ATP5A1 |  | HPA064755 | FGA |  | HPA060030 | IL6 |  | HPA011807 | POSTN |  | HPA013726 | TAS2R10 |
| HPA026856 | B4GALT5 |  | HPA019229 | FGL2 |  | HPA004932 | INS |  | HPA047815 | PPAP2A |  | HPA036629 | TAS2R10 |
| HPA060750 | B4GALT5 |  | HPA026682 | FGL2 |  | HPA046700 | IRF5 |  | HPA045049 | PSORS1C1 |  | HPA070201 | TAS2R10 |
| HPA067597 | B4GALT5 |  | HPA031092 | FKBP5 |  | HPA002267 | IRF8 |  | HPA050323 | PSORS1C1 |  | HPA015647 | TAS2R14 |
| HPA058284 | B4GALT6 |  | HPA031093 | FKBP5 |  | HPA002531 | IRF8 |  | HPA051817 | PSORS1C2 |  | HPA015957 | TAS2R14 |
| HPA062484 | B4GALT6 |  | HPA031095 | FKBP5 |  | HPA004627 | ISG15 |  | HPA056899 | PSORS1C2 |  | HPA042371 | TAS2R14 |
| Barebead | Negative control |  | HPA013392 | GAP43 |  | HPA061960 | KCNB2 |  | HPA061228 | PSORS1C2 |  | HPA062448 | TAS2R14 |
| HPA028477 | C1orf195 |  | HPA013603 | GAP43 |  | HPA004471 | KIT |  | HPA002834 | PTGS1 |  | HPA027064 | TAS2R3 |
| HPA045811 | C1orf195 |  | HPA015600 | GAP43 |  | HPA073252 | KIT |  | HPA001335 | PTGS2 |  | HPA056422 | TAS2R3 |
| HPA052116 | C1QB |  | HPA029730 | GATA3 |  | HPA061862 | KITLG |  | HPA054496 | PYCARD |  | HPA061025 | TAS2R3 |
| HPA046356 | C4A.C4B |  | HPA029731 | GATA3 |  | HPA070395 | KITLG |  | HPA019717 | RAB31 |  | HPA066998 | TAS2R3 |
| HPA046269 | C8G |  | HPA006667 | GBA |  | HPA019797 | KRT1 |  | RabbitIgG | Negative control |  | HPA043862 | TAS2R38 |
| HPA073328 | C8G |  | HPA019779 | GSTP1 |  | HPA030721 | LEP |  | HPA005839 | RASD2 |  | HPA054366 | TAS2R38 |
| HPA029577 | C9 |  | HPA019869 | GSTP1 |  | HPA030722 | LEP |  | HPA049152 | RETNLB |  | HPA028935 | TBX21 |
| HPA070709 | C9 |  | HPA003418 | GZMB |  | HPA057322 | LEP |  | HPA028081 | RGS18 |  | HPA046626 | TBX21 |
| HPA011652 | CCL11 |  | HPA012315 | HLADQA1 |  | HPA068565 | LEP |  | HPA028727 | RGS18 |  | HPA068747 | TBX21 |
| HPA042015 | CCL23 |  | HPA008338 | HMGCR |  | HPA018130 | LPA |  | HPA045780 | RGS18 |  | HPA047516 | TGFB1 |
| HPA063758 | CCL23 |  | HPA024035 | HPGDS |  | HPA060604 | LPA |  | HPA058436 | RGS18 |  | HPA073356 | TGFB1 |
| HPA010552 | CCL5 |  | MAB6487 | HPGDS |  | HPA072520 | LPA |  | HPA056183 | RNASE3 |  | HPA063582 | TGFB3 |
| HPA042290 | CCL5 |  | HPA050269 | HRG |  | HPA009431 | LRRN4 |  | HPA049098 | ROS1 |  | HPA053417 | TIMP1 |
| HPA053743 | CCL5 |  | HPA054598 | HRG |  | HPA009680 | LRRN4 |  | HPA053305 | ROS1 |  | HPA003829 | TLR2 |
| HPA014510 | CCR6 |  | HPA003901 | HSP90B1 |  | HPA031851 | MAGI1 |  | HPA037946 | RTKN2 |  | HPA051188 | TLR2 |
| HPA066394 | CCR6 |  | HPA008424 | HSP90B1 |  | HPA031852 | MAGI1 |  | HPA038446 | RTKN2 |  | HPA060231 | TLR2 |
| HPA046404 | CD163 |  | HPA049856 | HSP90B1 |  | HPA031853 | MAGI1 |  | HPA071940 | RTKN2 |  | HPA071546 | TLR2 |
| HPA051974 | CD163 |  | HPA050014 | HSP90B1 |  | HPA067854 | MAP2K3.MAP2K6 |  | HPA002881 | S100A12 |  | HPA050631 | TNF |
| HPA031566 | CD40 |  | HPA049525 | IFNG |  | HPA044497 | MAP2K3 |  | HPA003620 | S100A12 |  | HPA055037 | TNF |
| HPA031568 | CD40 |  | HPA053530 | IFNG |  | HPA006795 | MDC1 |  | HPA031828 | SCGB1A1 |  | HPA064998 | TNF |
| HPA002190 | CD55 |  | HPA063125 | IFNG |  | HPA006915 | MDC1 |  | HPA006225 | SELE |  | HPA012314 | TNFRSF11A |
| HPA045724 | CERS1 |  | HPA073359 | IFNG |  | HPA071976 | MDC1 |  | HPA057891 | SELE |  | HPA027728 | TNFRSF11A |
| HPA027262 | CERS2 |  | HPA007556 | IGF2 |  | HPA013949 | MGP |  | HPA065850 | SELE |  | HPA047976 | TNFRSF11A |
| HPA006092 | CERS3 |  | HPA007993 | IGF2 |  | HPA004920 | MMP1 |  | HPA067301 | SELE |  | HPA058613 | TNFRSF11B |
| HPA006102 | CERS3 |  | HPA027476 | IL10 |  | HPA008130 | MMP1 |  | HPA002655 | SELP |  | HPA027791 | TNFSF10 |
| HPA024356 | CERS3 |  | HPA051182 | IL10 |  | HPA031456 | MMP1 |  | HPA005990 | SELP |  | HPA045835 | TNFSF10 |
| HPA023621 | CERS4 |  | HPA071391 | IL10 |  | HPA054688 | MMP1 |  | HPA000927 | SERPINA1 |  | HPA054938 | TNFSF10 |
| HPA049826 | CERS4 |  | HPA065647 | IL10RA |  | HPA052343 | MMP10 |  | HPA001291 | SERPINA1 |  | HPA068318 | TNFSF10 |
| HPA070214 | CERS4 |  | HPA069086 | IL10RA |  | HPA053433 | MMP10 |  | HPA001292 | SERPINA1 |  | HPA046428 | TNNI3 |
| HPA006780 | CERS5 |  | HPA071295 | IL10RA |  | HPA051358 | MMP7 |  | HPA000893 | SERPINA3 |  | HPA063258 | TNNI3 |
| HPA026589 | CERS5 |  | HPA001886 | IL12A |  | HPA063662 | MMP7 |  | HPA042638 | SFTPA2.SFTPA1 |  | HPA036352 | TRAF3IP2 |
| HPA044683 | CERS6 |  | HPA041100 | IL12B |  | HPA073028 | MMP7 |  | HPA045752 | SFTPA2.SFTPA1 |  | HPA049742 | TRAF3IP2 |
| HPA063527 | CERS6 |  | HPA048230 | IL12B |  | HPA001238 | MMP9 |  | HPA049368 | SFTPA2.SFTPA1 |  | HPA069669 | TRAF3IP2 |
| HPA001143 | CFI |  | HPA018853 | IL13 |  | HPA063909 | MMP9 |  | HPA034820 | SFTPB |  | HPA004345 | TRIM33 |
| HPA024061 | CFI |  | HPA042421 | IL13 |  | HPA039412 | MOCOS |  | HPA062148 | SFTPB |  | HPA022816 | TSLP |
| AF2599 | CHI3L1 |  | HPA035737 | IL17A |  | HPA039888 | MOCOS |  | HPA010928 | SFTPC |  | HPA056350 | TSLP |
| HPA050947 | CHI3L1 |  | HPA045886 | IL17A |  | HPA047958 | MOCOS |  | HPA044582 | SFTPD |  | HPA005150 | TTR |
| HPA060867 | CHI3L1 |  | HPA052258 | IL17A |  | HPA061550 | MOCOS |  | HPA056768 | SFTPD |  | HPA024124 | UGCG |
| HPA072269 | CHI3L1 |  | HPA000437 | IL17RA |  | HPA021147 | MPO |  | HPA045191 | SGMS1 |  | HPA050554 | UGCG |
| MAB25991 | CHI3L1 |  | HPA001061 | IL17RA |  | HPA061464 | MPO |  | HPA063220 | SGMS1 |  | HPA014405 | UGT8 |
| HPA010115 | CHIT1 |  | HPA074140 | IL17RA |  | HPA047247 | MRPL43 |  | HPA015076 | SGMS2 |  | HPA065785 | UGT8 |
| HPA010575 | CHIT1 |  | HPA002837 | IL17RB |  | HPA055700 | MRPL43 |  | HPA064540 | SGMS2 |  | HPA001618 | VCAM1 |
| HPA074844 | CHIT1 |  | HPA052950 | IL17RB |  | HPA062877 | MRPL43 |  | HPA021125 | SGPL1 |  | HPA034795 | VCAM1 |
| HPA052634 | CMA1 |  | HPA072425 | IL17RB |  | HPA065191 | MRPL43 |  | HPA031108 | SLC11A1 |  | HPA034796 | VCAM1 |
| HPA006479 | CPA3 |  | HPA001410 | IL1B |  | HPA054563 | MS4A15 |  | HPA068540 | SLC11A1 |  | HPA069867 | VCAM1 |
| HPA006664 | CPA3 |  | HPA064606 | IL1B |  | HPA073616 | MS4A15 |  | HPA008549 | SLC22A2 |  | HPA048922 | ZNF688 |
| HPA043282 | CRISP3 |  | HPA068737 | IL1B |  | HPA045280 | NAPSA |  | HPA008567 | SLC22A2 |  |  |  |
