# Supplementary material for: Association of matrix metalloproteinase 7 and the alpha-chain of fibrinogen at baseline with response to methotrexate at 3 months in patients with early rheumatoid arthritis
Source: BMC Rheumatol. 2025 May 21;9:56. doi: 10.1186/s41927-025-00509-8 (PMC12093799; doi:10.1186/s41927-025-00509-8)
Supplement: Supplementary file 4 — Supplementary Material 4 [file 41927_2025_509_MOESM4_ESM.docx]

**Supplementary data**


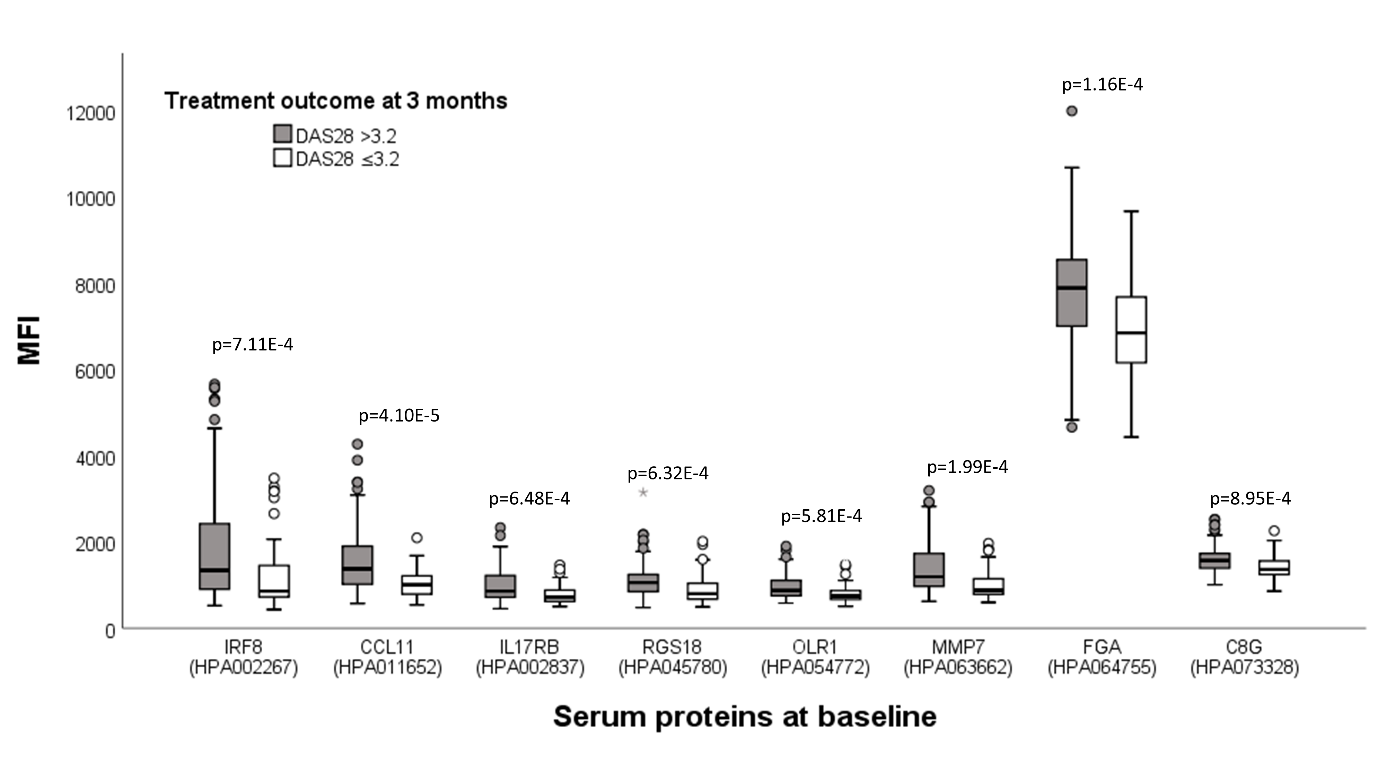


***Supplementary Figure S1. Baseline levels of median fluorescence intensity of serum proteins.*** *Distribution of MFI of detected proteins at baseline among patients with moderate/high DAS28 (grey boxes) and in LDA (white boxes) at 3 months. The boxes represent IQRs and whiskers – minimal and maximal values (excluding outliers) and dots represent outliers (values located further than 1.5 times the length of the IQR from the first and third quartiles, respectively).*


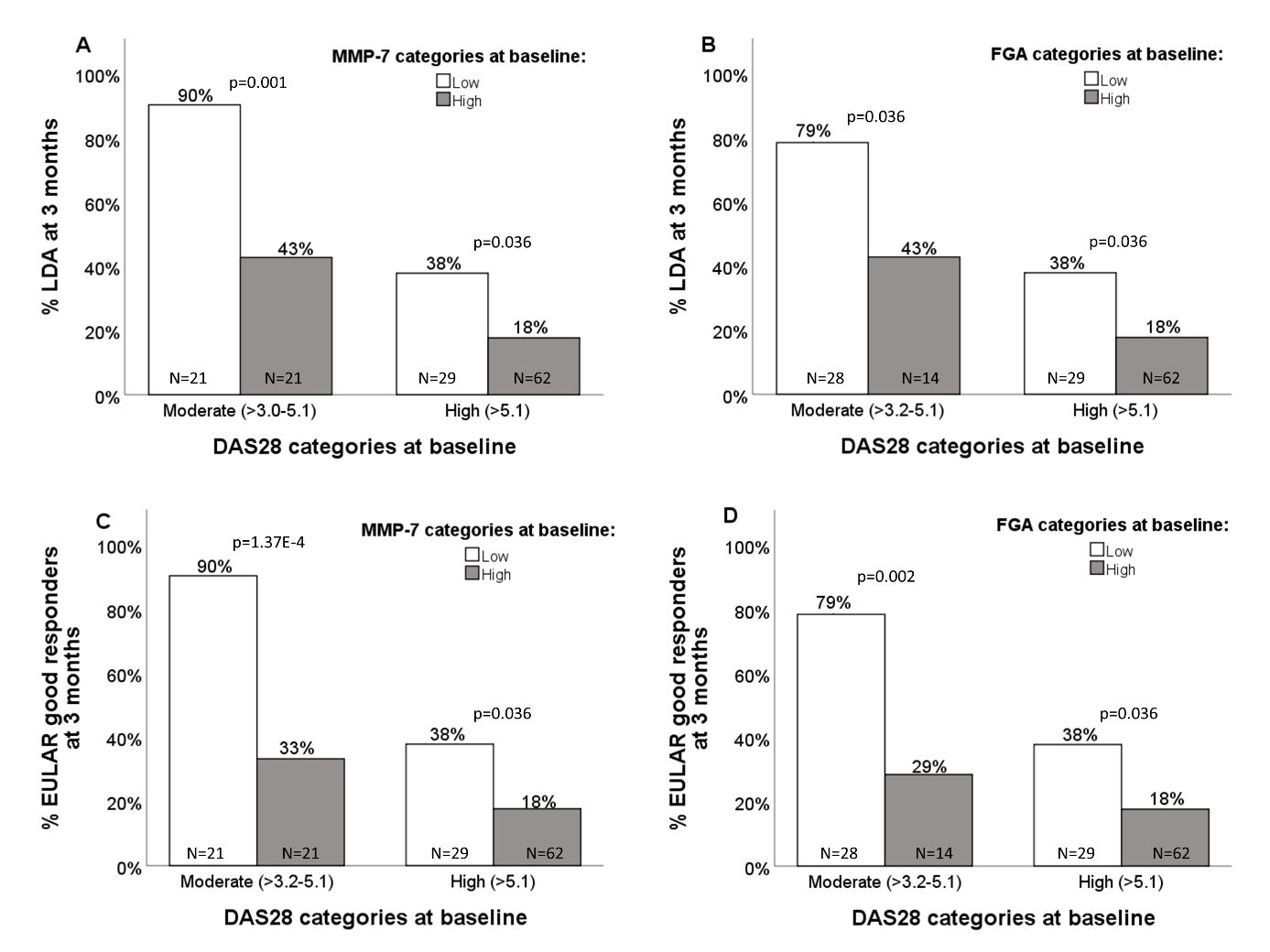


***Supplementary Figure S2. Proportion of patients with LDA or EULAR good response at 3 months among patients with low* versus *high levels of serum biomarkers within each group stratified by DAS28 at baseline.*** *Proportion of patients with LDA with low vs high MMP-7 (A) and low vs high FGA (B). Proportion of EULAR good responders with low vs high MMP-7 (C) and low vs high FGA (D). The white bars represent the proportion of patients with moderate DAS28 at baseline, and the gray bars represent the proportion of patients in the high DAS28 group at baseline.*

**Supplementary Table S1**. ID numbers and targets of all the antibodies (n=380) used in the suspension bead array.

|  | | | | | | | | | | | | | |
| --- | --- | --- | --- | --- | --- | --- | --- | --- | --- | --- | --- | --- | --- |
| **Abtibody ID** | **Targets** |  | **Abtibody ID** | **Targets** |  | **Abtibody ID** | **Targets** |  | **Abtibody ID** | **Targets** |  | **Abtibody ID** | **Targets** |
| HPA002265 | A2M |  | HPA054392 | CRISP3 |  | HPA005772 | IL1R2 |  | HPA047236 | NAPSA |  | HPA001823 | SMPD1 |
| HPA042506 | ACER1 |  | HPA048058 | CSF2 |  | HPA027598 | IL1R2 |  | HPA047744 | NAPSA |  | HPA018125 | SMPD2 |
| HPA013778 | ACER2 |  | HPA057404 | CSF2 |  | HPA007406 | IL1RL1 |  | HPA051835 | NCOA2 |  | HPA044442 | SMPD3 |
| HPA014092 | ACER2 |  | HPA071579 | CSF2 |  | HPA007917 | IL1RL1 |  | HPA069172 | NCOA2 |  | HPA058722 | SMPD3 |
| HPA038015 | ADCY2 |  | HPA040361 | CX3CL1 |  | HPA053829 | IL25 |  | HPA008422 | NFKB2 |  | HPA065535 | SMPD3 |
| HPA038483 | ADCY2 |  | HPA056729 | CX3CL1 |  | HPA068341 | IL25 |  | HPA050298 | NGF |  | HPA069383 | SMPD3 |
| HPA064436 | AGER |  | HPA073943 | CX3CL1 |  | HPA055164 | IL26 |  | HPA063135 | NGF |  | HPA001814 | SOD2 |
| HPA069474 | AGER |  | HPA014763 | DEGS1 |  | HPA061959 | IL26 |  | HPA003871 | NOS2 |  | HPA067587 | SPARCL1 |
| AntiAlbumin | Positive control |  | HPA014971 | DEGS1 |  | HPA069269 | IL26 |  | HPA038086 | NOS2 |  | HPA067641 | SPARCL1 |
| AntihumanIgG | Positive control |  | HPA057124 | DEGS1 |  | HPA046738 | IL2RA |  | HPA046487 | NOS2 |  | HPA022829 | SPHK1 |
| HPA046964 | ANXA2 |  | HPA021296 | DLG2 |  | HPA054622 | IL2RA |  | HPA007048 | NPSR1 |  | HPA023674 | SPHK1 |
| HPA061798 | ANXA2 |  | HPA021307 | DLG2 |  | HPA008412 | IL3 |  | HPA007106 | NPSR1 |  | HPA028761 | SPHK1 |
| HPA046715 | APOA1 |  | HPA023896 | DLG2 |  | HPA030770 | IL3 |  | HPA007489 | NPSR1 |  | HPA067105 | SPHK1 |
| HPA001352 | APOA4 |  | HPA027241 | ECM1 |  | HPA066598 | IL3 |  | HPA007976 | NPSR1 |  | HPA049062 | SPHK2 |
| HPA056395 | APOC3 |  | HPA066836 | ELANE |  | HPA022899 | IL33 |  | HPA050212 | NR3C1 |  | HPA057659 | SPHK2 |
| HPA065365 | APOC3 |  | HPA073774 | ELANE |  | HPA024426 | IL33 |  | HPA035619 | OLR1 |  | HPA065508 | SPHK2 |
| HPA065539 | APOE |  | HPA050507 | EPX |  | HPA052386 | IL33 |  | HPA035620 | OLR1 |  | HPA005562 | SPP1 |
| HPA068768 | APOE |  | HPA004826 | F7 |  | HPA007714 | IL4 |  | HPA050798 | OLR1 |  | HPA027541 | SPP1 |
| HPA001654 | APOH |  | HPA063808 | F7 |  | HPA063382 | IL4 |  | HPA054772 | OLR1 |  | HPA024330 | SPRR3 |
| HPA003732 | APOH |  | HPA035132 | FETUB |  | HPA070010 | IL4 |  | HPA047725 | ORM1.ORM2 |  | HPA044467 | SPRR3 |
| HPA047382 | ARFGAP1 |  | HPA035133 | FETUB |  | HPA065029 | IL5 |  | HPA057726 | ORM1.ORM2 |  | HPA044247 | SPTLC3 |
| HPA051019 | ARFGAP1 |  | HPA069860 | FETUB |  | HPA001325 | IL6 |  | HPA011325 | PDGFB |  | HPA048079 | SPTLC3 |
| HPA056273 | ARFGAP1 |  | HPA051370 | FGA |  | HPA044648 | IL6 |  | HPA011972 | PDGFB |  | HPA062197 | SPTLC3 |
| HPA040622 | ATP5A1 |  | HPA064755 | FGA |  | HPA060030 | IL6 |  | HPA011807 | POSTN |  | HPA013726 | TAS2R10 |
| HPA026856 | B4GALT5 |  | HPA019229 | FGL2 |  | HPA004932 | INS |  | HPA047815 | PPAP2A |  | HPA036629 | TAS2R10 |
| HPA060750 | B4GALT5 |  | HPA026682 | FGL2 |  | HPA046700 | IRF5 |  | HPA045049 | PSORS1C1 |  | HPA070201 | TAS2R10 |
| HPA067597 | B4GALT5 |  | HPA031092 | FKBP5 |  | HPA002267 | IRF8 |  | HPA050323 | PSORS1C1 |  | HPA015647 | TAS2R14 |
| HPA058284 | B4GALT6 |  | HPA031093 | FKBP5 |  | HPA002531 | IRF8 |  | HPA051817 | PSORS1C2 |  | HPA015957 | TAS2R14 |
| HPA062484 | B4GALT6 |  | HPA031095 | FKBP5 |  | HPA004627 | ISG15 |  | HPA056899 | PSORS1C2 |  | HPA042371 | TAS2R14 |
| Barebead | Negative control |  | HPA013392 | GAP43 |  | HPA061960 | KCNB2 |  | HPA061228 | PSORS1C2 |  | HPA062448 | TAS2R14 |
| HPA028477 | C1orf195 |  | HPA013603 | GAP43 |  | HPA004471 | KIT |  | HPA002834 | PTGS1 |  | HPA027064 | TAS2R3 |
| HPA045811 | C1orf195 |  | HPA015600 | GAP43 |  | HPA073252 | KIT |  | HPA001335 | PTGS2 |  | HPA056422 | TAS2R3 |
| HPA052116 | C1QB |  | HPA029730 | GATA3 |  | HPA061862 | KITLG |  | HPA054496 | PYCARD |  | HPA061025 | TAS2R3 |
| HPA046356 | C4A.C4B |  | HPA029731 | GATA3 |  | HPA070395 | KITLG |  | HPA019717 | RAB31 |  | HPA066998 | TAS2R3 |
| HPA046269 | C8G |  | HPA006667 | GBA |  | HPA019797 | KRT1 |  | RabbitIgG | Negative control |  | HPA043862 | TAS2R38 |
| HPA073328 | C8G |  | HPA019779 | GSTP1 |  | HPA030721 | LEP |  | HPA005839 | RASD2 |  | HPA054366 | TAS2R38 |
| HPA029577 | C9 |  | HPA019869 | GSTP1 |  | HPA030722 | LEP |  | HPA049152 | RETNLB |  | HPA028935 | TBX21 |
| HPA070709 | C9 |  | HPA003418 | GZMB |  | HPA057322 | LEP |  | HPA028081 | RGS18 |  | HPA046626 | TBX21 |
| HPA011652 | CCL11 |  | HPA012315 | HLADQA1 |  | HPA068565 | LEP |  | HPA028727 | RGS18 |  | HPA068747 | TBX21 |
| HPA042015 | CCL23 |  | HPA008338 | HMGCR |  | HPA018130 | LPA |  | HPA045780 | RGS18 |  | HPA047516 | TGFB1 |
| HPA063758 | CCL23 |  | HPA024035 | HPGDS |  | HPA060604 | LPA |  | HPA058436 | RGS18 |  | HPA073356 | TGFB1 |
| HPA010552 | CCL5 |  | MAB6487 | HPGDS |  | HPA072520 | LPA |  | HPA056183 | RNASE3 |  | HPA063582 | TGFB3 |
| HPA042290 | CCL5 |  | HPA050269 | HRG |  | HPA009431 | LRRN4 |  | HPA049098 | ROS1 |  | HPA053417 | TIMP1 |
| HPA053743 | CCL5 |  | HPA054598 | HRG |  | HPA009680 | LRRN4 |  | HPA053305 | ROS1 |  | HPA003829 | TLR2 |
| HPA014510 | CCR6 |  | HPA003901 | HSP90B1 |  | HPA031851 | MAGI1 |  | HPA037946 | RTKN2 |  | HPA051188 | TLR2 |
| HPA066394 | CCR6 |  | HPA008424 | HSP90B1 |  | HPA031852 | MAGI1 |  | HPA038446 | RTKN2 |  | HPA060231 | TLR2 |
| HPA046404 | CD163 |  | HPA049856 | HSP90B1 |  | HPA031853 | MAGI1 |  | HPA071940 | RTKN2 |  | HPA071546 | TLR2 |
| HPA051974 | CD163 |  | HPA050014 | HSP90B1 |  | HPA067854 | MAP2K3.MAP2K6 |  | HPA002881 | S100A12 |  | HPA050631 | TNF |
| HPA031566 | CD40 |  | HPA049525 | IFNG |  | HPA044497 | MAP2K3 |  | HPA003620 | S100A12 |  | HPA055037 | TNF |
| HPA031568 | CD40 |  | HPA053530 | IFNG |  | HPA006795 | MDC1 |  | HPA031828 | SCGB1A1 |  | HPA064998 | TNF |
| HPA002190 | CD55 |  | HPA063125 | IFNG |  | HPA006915 | MDC1 |  | HPA006225 | SELE |  | HPA012314 | TNFRSF11A |
| HPA045724 | CERS1 |  | HPA073359 | IFNG |  | HPA071976 | MDC1 |  | HPA057891 | SELE |  | HPA027728 | TNFRSF11A |
| HPA027262 | CERS2 |  | HPA007556 | IGF2 |  | HPA013949 | MGP |  | HPA065850 | SELE |  | HPA047976 | TNFRSF11A |
| HPA006092 | CERS3 |  | HPA007993 | IGF2 |  | HPA004920 | MMP1 |  | HPA067301 | SELE |  | HPA058613 | TNFRSF11B |
| HPA006102 | CERS3 |  | HPA027476 | IL10 |  | HPA008130 | MMP1 |  | HPA002655 | SELP |  | HPA027791 | TNFSF10 |
| HPA024356 | CERS3 |  | HPA051182 | IL10 |  | HPA031456 | MMP1 |  | HPA005990 | SELP |  | HPA045835 | TNFSF10 |
| HPA023621 | CERS4 |  | HPA071391 | IL10 |  | HPA054688 | MMP1 |  | HPA000927 | SERPINA1 |  | HPA054938 | TNFSF10 |
| HPA049826 | CERS4 |  | HPA065647 | IL10RA |  | HPA052343 | MMP10 |  | HPA001291 | SERPINA1 |  | HPA068318 | TNFSF10 |
| HPA070214 | CERS4 |  | HPA069086 | IL10RA |  | HPA053433 | MMP10 |  | HPA001292 | SERPINA1 |  | HPA046428 | TNNI3 |
| HPA006780 | CERS5 |  | HPA071295 | IL10RA |  | HPA051358 | MMP7 |  | HPA000893 | SERPINA3 |  | HPA063258 | TNNI3 |
| HPA026589 | CERS5 |  | HPA001886 | IL12A |  | HPA063662 | MMP7 |  | HPA042638 | SFTPA2.SFTPA1 |  | HPA036352 | TRAF3IP2 |
| HPA044683 | CERS6 |  | HPA041100 | IL12B |  | HPA073028 | MMP7 |  | HPA045752 | SFTPA2.SFTPA1 |  | HPA049742 | TRAF3IP2 |
| HPA063527 | CERS6 |  | HPA048230 | IL12B |  | HPA001238 | MMP9 |  | HPA049368 | SFTPA2.SFTPA1 |  | HPA069669 | TRAF3IP2 |
| HPA001143 | CFI |  | HPA018853 | IL13 |  | HPA063909 | MMP9 |  | HPA034820 | SFTPB |  | HPA004345 | TRIM33 |
| HPA024061 | CFI |  | HPA042421 | IL13 |  | HPA039412 | MOCOS |  | HPA062148 | SFTPB |  | HPA022816 | TSLP |
| AF2599 | CHI3L1 |  | HPA035737 | IL17A |  | HPA039888 | MOCOS |  | HPA010928 | SFTPC |  | HPA056350 | TSLP |
| HPA050947 | CHI3L1 |  | HPA045886 | IL17A |  | HPA047958 | MOCOS |  | HPA044582 | SFTPD |  | HPA005150 | TTR |
| HPA060867 | CHI3L1 |  | HPA052258 | IL17A |  | HPA061550 | MOCOS |  | HPA056768 | SFTPD |  | HPA024124 | UGCG |
| HPA072269 | CHI3L1 |  | HPA000437 | IL17RA |  | HPA021147 | MPO |  | HPA045191 | SGMS1 |  | HPA050554 | UGCG |
| MAB25991 | CHI3L1 |  | HPA001061 | IL17RA |  | HPA061464 | MPO |  | HPA063220 | SGMS1 |  | HPA014405 | UGT8 |
| HPA010115 | CHIT1 |  | HPA074140 | IL17RA |  | HPA047247 | MRPL43 |  | HPA015076 | SGMS2 |  | HPA065785 | UGT8 |
| HPA010575 | CHIT1 |  | HPA002837 | IL17RB |  | HPA055700 | MRPL43 |  | HPA064540 | SGMS2 |  | HPA001618 | VCAM1 |
| HPA074844 | CHIT1 |  | HPA052950 | IL17RB |  | HPA062877 | MRPL43 |  | HPA021125 | SGPL1 |  | HPA034795 | VCAM1 |
| HPA052634 | CMA1 |  | HPA072425 | IL17RB |  | HPA065191 | MRPL43 |  | HPA031108 | SLC11A1 |  | HPA034796 | VCAM1 |
| HPA006479 | CPA3 |  | HPA001410 | IL1B |  | HPA054563 | MS4A15 |  | HPA068540 | SLC11A1 |  | HPA069867 | VCAM1 |
| HPA006664 | CPA3 |  | HPA064606 | IL1B |  | HPA073616 | MS4A15 |  | HPA008549 | SLC22A2 |  | HPA048922 | ZNF688 |
| HPA043282 | CRISP3 |  | HPA068737 | IL1B |  | HPA045280 | NAPSA |  | HPA008567 | SLC22A2 |  |  |  |

**List of abbreviations**

alpha (α)-chain of fibrinogen (FGA)

anti-cyclic citrullinated peptide (anti-CCP)

area under the curve (AUC)

C-C motif chemokine ligand 11 (CCL11)

C-reactive protein (CRP)

complement component 8 γ-subunit (C8G)

Erythrocyte sedimentation rate (ESR)

Disease activity score 28 (DAS28)

early rheumatoid arthritis (eRA).

European League Against Rheumatism (EULAR)

Health assessment questionnaire (HAQ)

interferon regulatory factor 8 (IRF8)

interleukin-17B receptor (IL-17RB)

Low 28-joint disease activity score (LDA)

matrix metalloproteinase 7 (MMP-7)

median fluorescent intensity (MFI)

methotrexate (MTX)

oxidized low-density lipoprotein receptor 1 (OLR1)

Patient’s Global Assessment of Disease Activity (PatG)

regulator of G-protein signalling (RGS18)

Rheumatoid arthritis (RA)

Rheumatoid factor (RF)

Visual analogue scale (VAS)
